# Predicting Target DNA Sequences of DNA-Binding Proteins Based on Unbound Structures

**DOI:** 10.1371/journal.pone.0030446

**Published:** 2012-02-01

**Authors:** Chien-Yu Chen, Ting-Ying Chien, Chih-Kang Lin, Chih-Wei Lin, Yi-Zhong Weng, Darby Tien-Hao Chang

**Affiliations:** 1 Department of Bio-Industrial Mechatronics Engineering, National Taiwan University, Taipei, Taiwan; 2 Center for Systems Biology, National Taiwan University, Taipei, Taiwan; 3 Center for Biotechnology, National Taiwan University, Taipei, Taiwan; 4 Department of Computer Science and Information Engineering, National Taiwan University, Taipei, Taiwan; 5 Department of Electrical Engineering, National Cheng Kung University, Tainan, Taiwan; University of South Florida College of Medicine, United States of America

## Abstract

DNA-binding proteins such as transcription factors use DNA-binding domains (DBDs) to bind to specific sequences in the genome to initiate many important biological functions. Accurate prediction of such target sequences, often represented by position weight matrices (PWMs), is an important step to understand many biological processes. Recent studies have shown that knowledge-based potential functions can be applied on protein-DNA co-crystallized structures to generate PWMs that are considerably consistent with experimental data. However, this success has not been extended to DNA-binding proteins lacking co-crystallized structures. This study aims at investigating the possibility of predicting the DNA sequences bound by DNA-binding proteins from the proteins' unbound structures (structures of the unbound state). Given an unbound query protein and a template complex, the proposed method first employs structure alignment to generate synthetic protein-DNA complexes for the query protein. Once a complex is available, an atomic-level knowledge-based potential function is employed to predict PWMs characterizing the sequences to which the query protein can bind. The evaluation of the proposed method is based on seven DNA-binding proteins, which have structures of both DNA-bound and unbound forms for prediction as well as annotated PWMs for validation. Since this work is the first attempt to predict target sequences of DNA-binding proteins from their unbound structures, three types of structural variations that presumably influence the prediction accuracy were examined and discussed. Based on the analyses conducted in this study, the conformational change of proteins upon binding DNA was shown to be the key factor. This study sheds light on the challenge of predicting the target DNA sequences of a protein lacking co-crystallized structures, which encourages more efforts on the structure alignment-based approaches in addition to docking- and homology modeling-based approaches for generating synthetic complexes.

## Introduction

DNA-binding proteins are important to many biological processes in organisms. For example, transcription factors (TFs) activate or repress gene expression by using their DNA-binding domains (DBDs) to recognize specific nucleotide sequences in the genome. DNA sequences that can be recognized by the same DBD are usually characterized by a probabilistic model, called position weight matrix (PWM), to accommodate variability in sequences of TF-binding sites. Specifically, with the profile representation of TF binding sites (TFBSs), researchers can discover novel target genes regulated by known TFs. Therefore, accurate prediction of such target DNA sequences for DNA-binding proteins is an important step to understand many biological processes [1], [2], [3].

The most widely used technique of PWM inference for a TF is to collect a set of promoter sequences of genes known to be regulated by the TF and then detect frequently observed (over-represented) subsequences from the collection [4], [5], [6], [7], [8]. Such methods require sufficient sequences for pattern discovery, which are currently only available for a small amount of DNA-binding proteins. Similarly, the most promising technique to discover TF binding motifs, ChIP-seq [9], also has the limitation of requiring an antibody available for the TF. An alternative approach to predict PWMs is based on analyses of protein-DNA complex structures, which has been shown to perform well in telling which positions in a PWM should be more conserved and do not allow degeneration [10], [11], [12]. In this study we focused on the structure-based approaches to complement the predictions from sequence-based approaches. The later ones provide relatively limited information about how a DNA-binding protein binds to its target DNA. For example, when the interaction involves multiple proteins, sequence-based approaches cannot tell how many DBDs are required to interact with DNA.

Given a protein-DNA complex, the binding specificities of any DNA sequences to the proteins of the complex are first estimated by threading DNA sequences with a potential function. DNA sequences with high binding specificities are then summarized as a PWM. Existing potential functions of protein-DNA interactions are roughly categorized as physics-based [13], [14] and knowledge-based [12], [15], [16]. Physics-based potential functions focus on empirical energy components from physics, including electrostatics, hydrogen-bond, and van der Waals force [17], [18], [19], [20]. These potential functions have been applied to many important problems such as protein-DNA threading, docking decoy discrimination, and PWM prediction. Knowledge-based potential functions, on the other hand, adopt statistical components, such as the number of contacts and the distance distribution between the contacts, derived from known protein-DNA complexes. For PWM inference, knowledge-based potential functions have been shown to achieve similar prediction accuracy while saving much computation cost when compared to physics-based ones [12]. The contacts can be defined in residue level [15], [21] or atomic level [12], [16]. Residue-level knowledge-based potential functions have the advantages of requiring fewer protein-DNA structures to build the knowledgebase and processing less data when making predictions. However, they lose certain prediction accuracy due to ignoring the atomic-level structural variations. As the number of protein-DNA complexes has increased quickly in recent years, it is possible to construct atomic-level knowledge-based potential functions with sufficient sampling records. In 2005, Chang *et al.* proposed a potential function using 19 atom types [16], and in 2009, Xu *et al.* extended the set of atom types to 167 atom types for amino acids and 82 atom types for nucleotides [12].

Though these knowledge-based potential functions perform well on native complexes in predicting target DNA sequences, this success has not been extended to DNA-binding proteins lacking co-crystallized structures. In the 30 July 2011 release of Protein Data Bank (PDB) [22], only 403 out of about 1300 DNA-binding proteins have protein-DNA co-crystallized structures. This reveals an immediate need to develop PWM predictors for unbound protein structures. Such a predictor requires constructing a putative protein-DNA complex for the given unbound protein structure before PWM prediction. For this purpose, protein-DNA docking is one of the feasible ways to generate protein-DNA complexes but suffers high computational cost [23], [24]. To overcome this disadvantage, Gao and Skolnick recently employed an efficient way of generating protein-DNA complexes by structure alignment [21]. This structure alignment-based technique is adopted in this study to generate protein-DNA complexes to predict PWMs. Another technique that can be considered to generate putative protein-DNA complexes is homology modeling, which requires only the sequence of the query protein [11]. However, inferring target DNA sequences directly from protein sequence is beyond the scope of this study.

This study proposes a framework of PWM prediction based on unbound protein structures and investigates its feasibility and challenges. Given a query protein structure and a template complex, the proposed method conducts structure alignment to generate superimposed protein-DNA complexes. Based on the protein-DNA complex, an atomic-level knowledge-based potential function is employed to predict PWMs to which the query protein can bind. To the best of our knowledge, this study is the first work of inferring target DNA sequences from unbound protein structures based on structure alignment technique. We compiled a benchmark of seven DNA-binding proteins which have annotated PWMs and structures of both DNA-bound and unbound forms. Requesting both forms is for comparing the performance of the potential function applied on the native and synthetic complexes. The experimental results show that though the performance based on the synthetic complexes generated by the proposed framework is worse than that based on the native complexes, it is better than that simply based on the homologous complexes. Potential reasons behind the performance difference between our synthetic complexes and the native ones were further investigated by progressively adjusting the quality of the synthetic complexes towards the condition mimicking the native complexes. Finally, the synthetic complexes based on structure alignment were compared with those based on protein-DNA docking. The results show that the proposed framework was comparable to that based on docking and is much more efficient. The kernel of the proposed method, which makes prediction based on a given pair of an unbound structure (query) and a user-specified complex (template), is released along with this study as a Linux executable (http://mbi.ee.ncku.edu.tw/res/Chen_2011/).

## Results/Discussion

**Figure 1:**
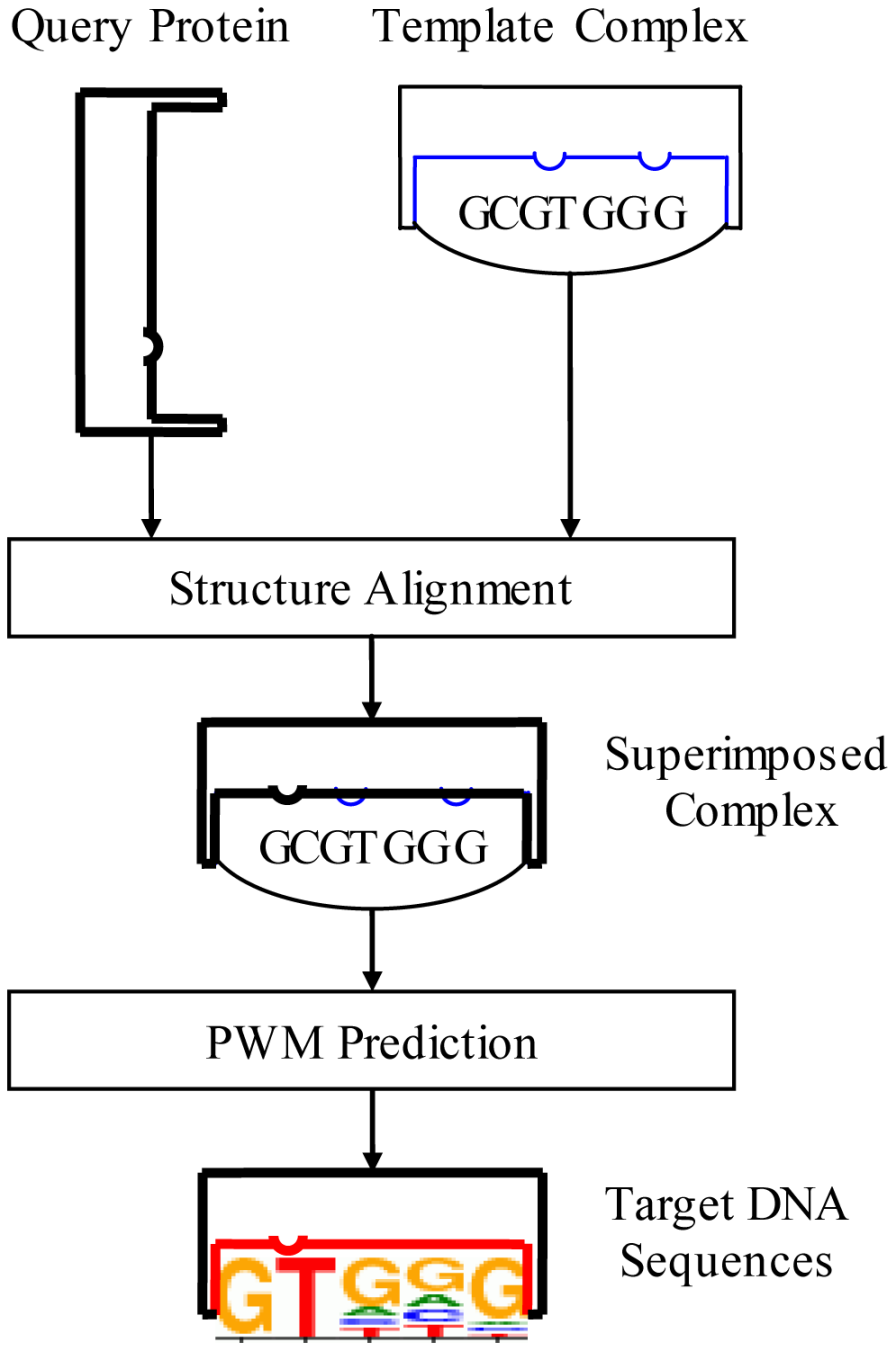
The workflow of the proposed method. The query protein is superimposed onto the specified template structure and then the PWM prediction is performed on the superimposed protein-DNA complex based on a knowledge-based potential function considering atomic contacts.

Figure 1 shows the workflow of the proposed method. Given an unbound query protein and a template complex, the proposed method generates synthetic protein-DNA complex structures for PWM prediction using structure alignment, where the query protein is superimposed onto the template structure (‘Superimposed Complex’ in Figure 1). This is achieved by applying the rotation matrix reported by the structure alignment program. PWM prediction is then performed on the superimposed protein-DNA complex based on an all-atom model, which is a knowledge-based potential function considering atomic contacts. See the ‘Methods’ section for the details of a) constructing the superimposed complex based on the given query and template structures and b) the employed all-atom model.

### Validation set

To evaluate the performance of the proposed framework, we first considered the 20 annotated PWMs and the corresponding native protein-DNA complexes from the study of Morozov *et al.* [10]. The structure with discontinuous dsDNA (1IHF) was excluded as in the study of Xu *et al.* [12]. Since the proposed method requires an unbound structure of the query protein and a native complex from any of its homologues as the template, we must require each of the 19 potential test cases to further pass the following criterion: to have an unbound structure in PDB which yields at least one qualified alignment to a DNA-bound structure of another protein.

For each of the 19 proteins, we first checked if it has an unbound structure that can be used as a query in the proposed method. Only 12 of them have unbound structures in the 30 July 2011 release of PDB. Each unbound structure was then compared to the protein chains of all the protein-DNA complexes in PDB by using PSI-BLAST [25] for measuring the sequence similarity and by TM-align [26] for the structure similarity. If the significance of sequence similarity passes the condition of e-value<0.001 and the structure alignment score, TM-score [27], is greater than 0.5, the qualified complex was collected in the set of potential template complexes. Here, we required that a template structure must satisfy the following criteria: a) it is an X-ray structure with resolution better than 3.0 Å, b) the DNA molecule has ≥6 paired bases and has less than 30% non-paired bases, c) the protein chain has ≥5 contact residues (residues within 4.5 Å to the DNA molecule) and d) the protein chain has ≥40 residues. In this study, the query-template pair with the highest TM-score for each of the potential test cases was chosen for PWM prediction. In the end, six proteins were used as test cases and the other 13 proteins that do not satisfy the above criterion were used for tuning the parameters of the all-atom model (Table 1).

**Table 1:** The validation set used in this study.

PDB	Entry name^a^	Protein
Seven proteins used as the queries		
6CRO	RCRO_LAMBD	Regulatory protein cro
1MSE	MYB_MOUSE	Transcriptional activator Myb
1MNN	NDT80_YEAST	Meiosis-specific transcription factor NDT80
1YRN	MATA1_YEAST	Mating-type protein A1
1TRO	TRPR_ECOLI	Trp operon repressor
1RUN	CRP_ECOLI	Catabolite gene activator
2O61^b^	NFKB1_HUMAN	Nuclear factor NF-kappa-B p105 subunit
13 complexes used for tuning the parameters of the all-atom model		
1AAY	EGR1_MOUSE	Early growth response protein 1
1B8I^c^	UBX_DROME	Homeotic protein ultrabithorax
	EXD_DROME	Homeobox protein extradenticle
2DRP	TTKB_DROME	Protein tramtrack, beta isoform
1FJL	PRD_DROME	Segmentation protein paired
1GCC	ERF1A_ARATH	Ethylene-responsive transcription factor 1A
1GXP	PHOB_ECOLI	Phosphate regulon transcriptional regulatory protein phoB
1J1V	DNAA_ECOLI	Chromosomal replication initiator protein dnaA
1LMB	RPC1_LAMBD	Repressor protein CI
1MJ2	METJ_ECOLI	Met repressor
2PUC	PURR_ECOLI	HTH-type transcriptional repressor purR
1R0O	USP_DROME	Protein ultraspiracle
1YSA	GCN4_YEAST	General control protein GCN4
1YUI	GAGA_DROME	Transcription factor GAGA

a UniProt entry name.

b not used in the study of Morozov *et al.* [10].

c containing two chains of different proteins.

In addition to the test cases collected from the study of Morozov *et al.*, this study attempted to enlarge the test data by collecting more annotated PWMs from the TRANSFAC database [28]. The public version of TRANSFAC contains 398 annotated PWMs for 133 UniProt [29] entry names. However, due to the limited overlap between the list of proteins with annotated PWMs and the list of proteins with both unbound and available templates, only one more test case (NFKB1_HUMAN) was added, as shown in Table 1.

### Evaluating PWM prediction using unbound protein structures

**Figure 2:**
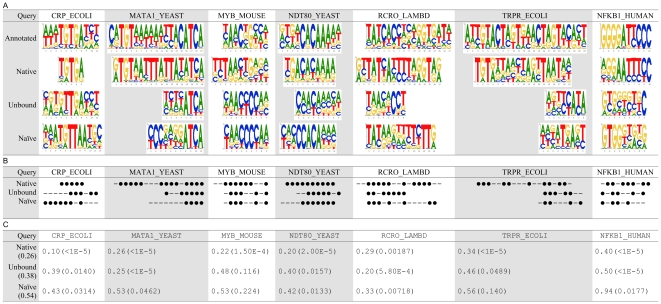
Predictions by the proposed method on the seven test cases. The predictions of the proposed method are denoted as ‘Unbound’, in comparison with the annotated PWMs (‘Annotated’), the predicted PWMs based on native complexes (‘Native’) and the complexes of homologues (‘Naïve’). (A) PWMs. (B) A position is marked as ‘•’ if its most favorable base type was correctly predicted, or marked as ‘–’ otherwise. (C) *Ψ*-scores and the corresponding p-values. The value within the parentheses of the first column indicates the average *Ψ*-score.

The detailed predictions on the seven test proteins using their unbound structures are provided in Figure 2 (denoted as ‘Unbound’), in comparison with the annotated PWMs provided by [10] (denoted as ‘Annotated’) and the predicted PWMs based on their native complexes (denoted as ‘Native’). The involved PDB entries are listed in Table 2. In this study, the *Ψ*-score used in [10] was employed to evaluate the performance of the proposed method. *Ψ*-score is the average of the Kullback-Leibler divergences across all positions, and was adopted to evaluate the consistency between the predicted and annotated weight scores for all base types. The definition of the *Ψ*-score is provided as follows:
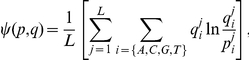
where 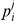 and 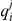 are predicted and annotated weight scores, respectively, for base type *i* at position *j*, and *L* is the length of the binding site in base pairs. A smaller number on the *Ψ*-score implies a higher degree of consistency between two PWMs. To measure the significance of a *Ψ*-score, 100,000 dummy PWMs with the same length as the predicted PWM were randomly generated to estimate the null distribution of *Ψ*-scores to the annotated PWM and the p-value of the *Ψ*-score of the predicted PWM.

**Table 2:** The PDB entries used in this study.

Entry name	Native^a^	Query^b^	Template^c^
CRP_ECOLI	1RUN	2GZW:A	3E6C:C
MATA1_YEAST	1YRN	1MH3:A	2HOS:A
MYB_MOUSE	1MSE	1GV2:A	1W0T:A
NDT80_YEAST	1MNN	1M6U:A	1HJC:A
RCRO_LAMBD	6CRO	2A63:A	3CRO:R
TRPR_ECOLI	1TRO	1MI7:R	1YSA:D
NFKB1_HUMAN	2O61	1NFI:D	1HJC:A

a native complexes of the corresponding proteins.

b unbound structures of the corresponding proteins.

c native complexes of different proteins used as the templates.

The proposed framework achieved 0.38 *Ψ*-score in average, which was worse than that (0.26 *Ψ*-score) based on the native complexes. Even though the average *Ψ*-score of using unbound structures is worse than that of using native complexes, the difference is not significant (the p-value of paired Wilcoxon signed-rank test [30] is 0.078). We also compared the proposed method with a naïve approach that predicts PWMs directly based on the homologues' native complexes of the query structures using the all-atom model. Namely the naïve method uses the query unbound structure to search the homologous bound structures but not replace the protein in the homologous structure with the query structure. This approach is denoted as ‘Naïve’ in Figure 2, where the homologous bound structure of each case used for prediction was the corresponding template structure in Table 2. The average *Ψ*-score of the naïve approach is 0.54, and the p-value of paired Wilcoxon signed-rank test between the proposed method and the naïve approach is 0.016.

It is observed in Figure 2 that the widths of the predicted PWMs are usually shorter than the annotated ones. This is because that the proposed method can only infer the target DNA sequences physically contactable by the query protein in the superimposed complexes. Protein-DNA interactions sometimes require multiple protein chains to participate in. Since the query unbound structure is simply one of them, the predicted PWM might be shorter than i) that based on native complexes which contain the complete set of protein chains and ii) the annotated PWMs derived from experiments or conserved promoter sequences.

We also compared the predictions on the six test cases from [10] to those of applying different potential functions [10], [12] on native complexes (Table 3). Table 3 shows that the predictions of using native complexes generally outperforms that of using synthetic complexes constructed based on the unbound structures and the selected templates. The results shown in Table 3 and Figure 2 both reveal the potential of conducting PWM prediction for DNA-binding proteins based on unbound structures, though the accuracy degrades when synthetic complexes were used instead of native complexes. It is reasonably speculated that the performance difference was due to structural variations between the native complexes and the synthetic complexes generated by structure alignment followed by superposition. The next subsection lists three types of structural variations that presumably influence the prediction accuracy and provides further analyses to investigate these structural variations. The first considers the variation on the binding position or orientation caused by structure alignment. In other words, the complexes generated by structure alignment might have structural variations deviated from crystallized complexes. The second one is the structural variation due to sequence difference. That is, the binding position or orientation might have variations on two different protein sequences, even though their structures are similar. The third structural variation we considered is the conformational change of proteins from the unbound to bound form.

**Table 3:** Predictions using unbound structures compared with those using native complexes.

	Native										Unbound^d^
PDB	Random^a^	Contact^b^	Static^b^	Dynamics^b^	DDNA^a^	FIRE^a^	vFIRE^a^	cFIRE^a^	vcFIRE^a^	All-atom^c^	All-atom^c^
6CRO	0.47	0.07	0.10	0.21	0.26	0.10	0.10	0.09	0.10	0.29	0.20
1MSE	0.55	0.26	0.24	-	0.66	0.21	0.21	0.10	0.09	0.22	0.48
1MNN	0.68	0.14	0.12	0.20	0.46	0.25	0.25	0.22	0.22	0.20	0.40
1YRN	0.73	0.20	0.26	0.36	0.20	0.33	0.33	0.28	0.30	0.26	0.25
1TRO	0.71	0.30	0.31	0.39	0.42	0.42	0.42	0.42	0.43	0.34	0.46
1RUN	0.51	0.10	0.17	0.38	0.55	0.23	0.24	0.23	0.19	0.10	0.39
Average	0.61	0.18	0.20	0.31	0.43	0.26	0.26	0.22	0.22	0.24	0.36
Sd	0.11	0.09	0.08	0.09	0.17	0.11	0.11	0.12	0.13	0.08	0.11

a data from Xu *et al.* [12].

b data from Morozov *et al.* [10].

c our implementation, which is a variation of FIRE.

d the unbound structures and the corresponding templates used were listed in Table 2.

### Evaluating robustness of the proposed method against structural variations

For the first structural variation from the alignment, we want to know if the proposed method yields stable predictions when the protein structure in a native complex is replaced by a protein structure from another native complex of the same protein using structure alignment. Namely, the query protein, which is also a bound structure, is superimposed to another complex of the same protein. This design aims to eliminate the influence of the other two structural variations. For this purpose, we grouped protein-DNA complexes in PDB by the UniProt entry names of the protein chains. Protein chains in complexes with multiple protein chains were excluded. In the end, we have 38 PDB chains and 74 query-template pairs over eight entry names, where each entry name has 4–6 PDB chains. Table 4 shows the results of the analysis regarding the first structural variation. All the values of *Ψ*-score are quite small. These results reveal an important observation that the proposed method is robust to the structural variations among native complexes of the same protein determined from different experiments as well as the variations due to structure alignment.

**Table 4:** Performance on identical protein using different native complexes.

Entry Name	Number of chains	Number of pairs	*Ψ*-score^a^
DN71_SULAC	4	6	0.02±0.01
EGR1_MOUSE	4	6	0.05±0.03
P84131_BACST	4	6	0.08±0.05
POL_MLVMO	4	6	0.01±0.01
DPO1_BACST	5	10	0.00±0.00
UNG_HUMAN	5	10	0.11±0.12
FPG_LACLC	6	15	0.00±0.00
MTH1_HAEHA	6	15	0.04±0.03
Overall	38	74	0.04±0.06

a Mean ± standard variation.

**Figure 3:**
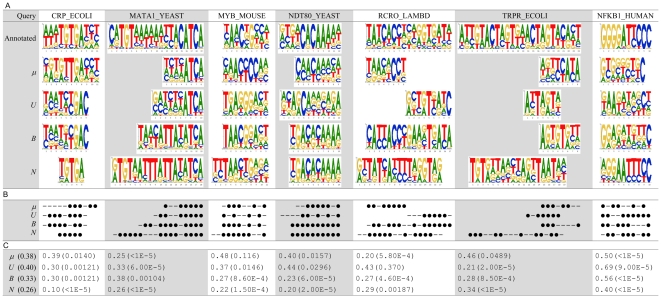
Predictions using different complexes. *μ*: the proposed method. *U*: the second synthetic complex that eliminates the second type of structural variation. *B*: the third synthetic complex that eliminates the second and third types of structural variation. *N*: native complexes. (A) PWMs. (B) A position is marked as ‘•’ if its most favorable base type was correctly predicted, or marked as ‘–’ otherwise. (C) *Ψ*-scores and the corresponding p-values. The value within the parentheses of the first column indicates the average *Ψ*-score.

To investigate the second structural variation due to sequence difference, we prepared the second synthetic complex (*U*) where the template is a complex of the query protein itself—instead of a complex of a different protein—for each query in the validation set (Table 5). Figure 3 shows that using this set achieved an average *Ψ*-score of 0.40, which is close to that of using a different protein (0.38). The p-value of the paired Wilcoxon signed-rank test on the *Ψ*-scores of these two sets (*μ* and *U*) is 1. Namely, there was no apparent improvement observed when we eliminated this type of structural variation in the prediction framework. This suggests that the all-atom model with the proposed framework can tolerate the structural differences between different proteins that share similar structures.

**Table 5:** The three synthetic complexes employed in the analysis of structural variations.

Synthetic complex	Query protein	Template protein	Denoted as
The first synthetic complex (the proposed synthetic complex)	Unbound	Different to the query	*μ*
The second synthetic complex	Unbound	Identical to the query	*U*
The third synthetic complex	Bound	Identical to the query	*B*

To investigate the third structural variation of the conformational change between unbound and bound forms, we prepared the third synthetic complex (*B*) by replacing the query of the second synthetic complex with a bound structure for each query in the validation set (Table 5). Using this set achieved *Ψ*-score of 0.33 (Figure 3). This performance was better than those using unbound queries and close to those using native complexes. The performance gap after eliminating this type of structural variation indicates that the structural variation of the conformational change is the most critical structural variation to the prediction accuracy. These results reveal that the proposed framework is more sensitive to the structural changes between unbound and bound conformations than those between two homologous structures. Hence, if we want to construct PWMs directly from an unbound structure with higher accuracy, the first priority of the next step is to overcome the unbound-to-bound conformational change.

In Table 6, we provided with more details about the structural changes upon DNA-binding of the seven test cases based on the same query (unbound) and template (bound) structures as the second synthetic complex (*U*). Two special structural transitions, transitions of secondary structures (SSE) and disorder-to-order (D2O) transitions discussed in a recent study [31], were in particular examined here in addition to the root-mean-square deviations (RMSDs) between a pair of structures. In this table, we observed that structural variations are not necessarily accompanied with structural transitions. For example, the used structures for MYB_MOUSE have the largest RMSD (2.88) but have neither SSE nor D2O transitions. The structures used for NDT80_YEAST have 25 D2O transitions but a small RMSD (0.72).

**Table 6:** Structural transitions upon DNA-binding.

Entry name	Unbound	Bound	SSE^a^	D2O^b^	RMSD^c^	*Ψ*-score
CRP_ECOLI	2GZW:C	2CGP:A	0	0	0.73	0.30
MATA1_YEAST	1MH3:A	1YRN:A	0	0	0.90	0.33
MYB_MOUSE	1GV2:A	1H89:C	0	0	2.88	0.37
NDT80_YEAST	1MN4:A	2EUX:A	0	25	0.72	0.44
RCRO_LAMBD	2OVG:A	6CRO:A	0	0	0.83	0.43
TRPR_ECOLI	1JHG:A	1TRO:C	0	0	1.02	0.21
NFKB1_HUMAN	1NFI:D	2O6I:B	0	0	0.50	0.69

a SSE: transition of secondary structure.

b D2O: disorder-to-order transition.

c RMSD: root mean square deviation.

### Comparison with predictions based on complexes generated by docking

The above experiments were designed to evaluate the quality of the synthetic complexes under the proposed framework. This section, on the contrary, compares the prediction performance of using the synthetic complexes obtained by the proposed framework to that obtained by protein-DNA docking. Here we adopted the ZDOCK package (version 2.3.1) to perform protein-DNA docking. The protein structure was prepared using the query structures and the DNA was prepared using the bound DNA structures of the templates listed in Table 2. In the proposed framework, a template of protein-DNA complex is employed to facilitate the generation of synthetic complexes. In other words, the DNA-binding residues of the protein were learned from an existing protein-DNA complex. For a fair comparison, the same information was exploited here to rank models generated by ZDOCK. We assigned the highest score to the synthetic complex that reserves the largest set of the expected contact residues. Complexes reserving the same number of contact residues kept the same order suggested by ZDOCK. Based on the new scoring strategy, the top 20 complexes of the 2000 ZDOCK predictions (here 2000 was set according to the ZDOCK manuscript) were used to perform PWM prediction. Finally, the predicted PWM with the best *Ψ*-score to the annotated PWM was reported here. The process of using the *Ψ*-score to select PWM, note that it favors ZDOCK, was adopted because we observed that the highest scored complexes resulted in extremely bad PWMs, which were difficult to be aligned to the annotated ones in all tests (data not shown).

**Figure 4:**
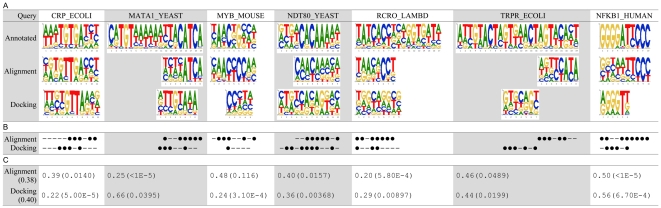
Comparison with predictions of using docking to construct synthetic complexes. The predictions based on the proposed alignment-based approach to construct synthetic complexes are denoted as ‘Alignment’, while those of ZDOCK are denoted as ‘Docking’. (A) PWMs. (B) A position is marked as ‘•’ if its most favorable base type was correctly predicted, or marked as ‘–’ otherwise. (C) *Ψ*-scores and the corresponding p-values. The value within the parentheses of the first column indicates the average *Ψ*-score.

Figure 4 shows the comparison of using the proposed framework (denoted as ‘Alignment’ in Figure 4) and using the protein-DNA docking to generate the protein-DNA complex for PWM prediction. Using the docked complexes achieved an average *Ψ*-score of 0.40, worse than the proposed method. We observed that the PWMs generated by the proposed method and docking have their own advantages in different positions even though the same queries and templates were used. For example, for the center five positions (‘TGTGA’), which are more conserved than the flanking positions in the annotated PWM of CRP_ECOLI, the docking's PWM only missed the fourth position. On the other hand, our PWM correctly predicted the fourth position but missed the first two positions. On the test case NDT80_YEAST, the docking's PWM correctly predicted the six positions (2–3 and 5–8) on the left part while our PWM correctly predicted the six positions (6–10 and 12) on the right part of the annotated PWM. For TRPR_ECOLI, the docking's PWM has no overlap with our PWM, but both of them are generally correct since the interaction actually involve two identical protein chains. In summary, the docking's and our PWMs both made good predictions on some test cases though on different positions. Regarding the efficiency issue, ZDOCK takes more than an hour for the seven test cases, which is much longer than that (less than ten seconds) of the proposed method based on structure alignment.

The complementary phenomenon of the docking's and our predictions might be due to the structural variations—mainly from unbound to bound—discussed in the previous subsection. The query structures must undergo some conformational change so that they can fit the DNA molecules well. However, both the proposed framework and the adopted docking strategy regarded the query structures as rigid bodies. It might happen that one end of the binding site of the query structure perfectly fit the DNA but the other end was ‘seesawed’ out its best position.

### Discussion and concluding remarks

It was discussed in the study of Dan *et al.* [31] that conformational changes were commonly observed in DNA-binding proteins. To understand how common the conformational changes are present in protein-DNA interactions and how large the changes are usually observed, we further collected available structure pairs of unbound and bound states for DNA-binding proteins from the PDB database. Since a protein may have multiple unbound-bound structure pairs, we adopted a strict criterion that a protein has transitions if at least one of the associated unbound-bound structure pair has transitions. The definition of transitions between a structure pair is identical to that of Dan *et al.*'s work (the DSSP program was used to assign secondary structures and only segments in which the same transition was consistent for at least five consecutive residues were considered). The results show 40.2% of the 132 proteins underwent SSE transitions (changes on secondary structure) and 53.8% underwent D2O (disorder-to-order) transitions. The high ratios concur with the points of Dan *et al*.

On the other hand, it is observed that the RMSD values were not that large, *i.e.*, all structure pairs were less than 4 Å (data not shown). If the criterion ‘RMSD≤2 Å’, a rigorous threshold in general, is considered to indicate small structural variation, 93.2% proteins have at least one structure pair with small structural variation. In Table 6, we found that the ratio of proteins underwent SSE (0.0%) and D2O (14.3%, one among the seven test cases) transitions were much lower than those of the overall distribution (40.2% SSE and 53.8% D2O transitions). The major difference between Table 6 and the analysis in this section is that in Table 6 the structure pair was selected by the structure alignment score. This suggests that in practice using the best structure alignment score helps to find structure pairs with few transitions for PWM prediction. If the structure pair with the best RMSD is chosen to investigate the conformational changes of a protein upon binding DNA, we found that ratios of proteins which underwent SSE and D2O transitions dropped to 13.8% and 39.4%, respectively. These results suggest that the proposed method will benefit the study of a large quantity of DNA-binding proteins with only unbound structures in the PDB database.

**Figure 5:**
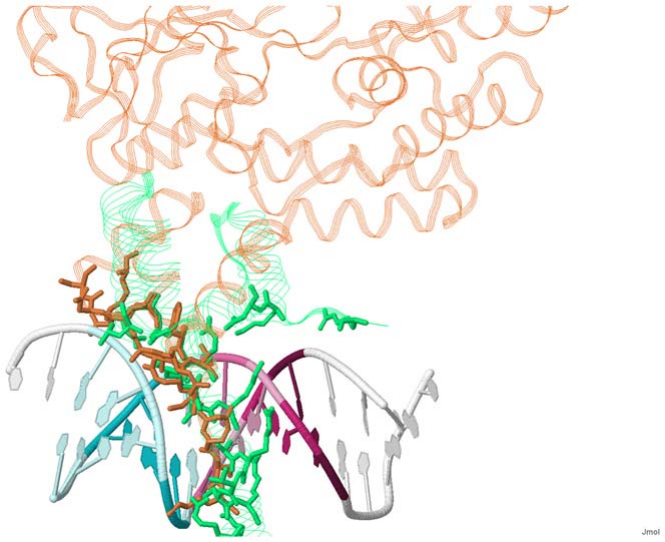
An example where the template has a low structure similarity to the query. This case demonstrates that using less similar templates still has the potential to produce satisfied results. This figure contains two proteins that share similar DNA-binding interface but have low global structure similarity (TM-score = 0.38). The *Ψ*-score of the predicted PWM to the annotated PWM using the orange protein (1MH3:A, MATA1_YEAST) as the query and the green protein (1SKN:P, SKN1_CAEEL) as the template is 0.18. Contact residues on both protein structures are plotted in *sticks* presentation.

To shift the problem of structure-based PWM prediction from native complexes to unbound protein structures, the most challenging issue might be constructing a reliable synthetic protein-DNA complex on which physics- or knowledge-based scoring functions can be applied to perform prediction. Regarding this issue, this study concludes that structure alignment can serve as an option when complexes containing bound structures similar to the query protein exist. Though currently we used the template with the highest structure similarity to generate the synthetic complex, it is observed in many cases that templates with a low structure similarity also have the potential to produce satisfied results, as exemplified in Figure 5.

**Figure 6:**
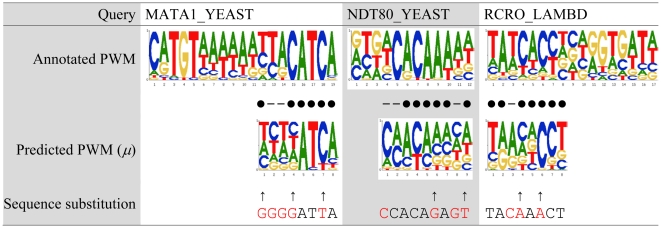
Demonstration of base substitution. This case demonstrates the ability of the employed all-atom potential function to replace the base types when the native DNA sequence in the selected template is not the same as the target DNA sequence to that the query protein can bind. A position is marked as ‘•’ if its most favorable base type was correctly predicted, or marked as ‘–’ otherwise. In addition, the symbol ‘↑’ stands for a successful substitution. The sequence shown is the DNA sequence in the selected template, where red nucleotides indicate the positions of which the bases are different to the most favorable base types in the annotated PWMs.

Two concluding remarks are provided here. The DNA sequence in the selected template is probably not the native DNA sequence to which the query protein can bind. Thus the ability of the adopted potential function to handle the mutations of DNA sequences embedded in the synthetic complex is critical to the success of the proposed framework. Regarding this issue, we concluded that the selected atomic knowledge-based potential function is generally able to predict the most favorable base type without being affected by the original sequence present in the synthetic complex. Three examples are shown in Figure 6 to illustrate this observation. Another important issue related to the development of structure-based methods is their applicability. In the PDB release of July 30, 2011, there are 114 DNA-binding proteins that do not have native complexes but have unbound structures with potential templates from homologues available. The definition of a pair of unbound structure and the potential template is e-value<0.001 for the sequence alignment and TM-score >0.5 for the structure alignment. Currently the public version of TRANSFAC database contains 398 annotated PWMs for 133 proteins, most of which were determined via sequence-based methods. However, the overlap between the 114 DNA-binding proteins, which are the targets of this study, and the 133 proteins with known PWMs is only 16. This small overlap concurs with the fact that the currently curated PWMs were majorly contributed by sequence-based methods. This also reveals the distinctness and potential of structure-based methods, since up to now an abundance of structure information has not been widely exploited to enhance our understandings about the interactions between DNA-binding proteins and their binding sites.

### Conclusion

Accurate construction of binding sequences for DNA-binding proteins is an important step for studying protein-DNA interactions. This study proposes a novel prediction framework and shows the possibility of predicting target DNA sequences of DNA-binding proteins directly from their unbound forms. Several factors that might affect the prediction power of the proposed method are examined and discussed. The experiments conducted in this study encourage more efforts on the structure alignment-based approaches as well as raise the challenges of PWM prediction using unbound protein structures for future studies.

## Methods

In this section, we first describe how structure alignment is performed to generate appropriate superimposed complexes for the query protein. Next, we introduce the potential function used for PWM prediction.

### Constructing superimposed complexes

As shown by the ‘Superimposed Structure’ in Figure 1, the query protein is superimposed onto the template structure. This is achieved by applying the rotation matrix reported by the structure alignment tool, TM-align [26]. We removed the original protein chains in the template and appended the transformed coordinates of the query structure into the template structure to generate a superimposed complex for PWM prediction.

### The potential function for PWM prediction

The objective of this study is to replace the protein structure in native complex structures with the query protein structure. A scoring function that takes the amino acid types into consideration is desired. We implemented a variation of the FIRE potential function, named as ‘all-atom model’ in the context, described by [12] for this purpose. FIRE is a succinct knowledge-based potential function that considers interactions between all atom types. Different knowledge-based potential functions adopted different reference states. The reference state used in FIRE and in this study is an averaged reference state based on a collection of protein-DNA complexes, namely *knowledgebase*. Among the series of all-atom scoring functions presented in [12], FIRE has the advantage of easy implementation and is shown to be generally as good as two of its extended functions, cFIRE and vcFIRE, in predicting PWMs.

To construct the knowledgebase, we first denote the number of pairs of atom types *i* and *j* with the distance falling within a specified range (*r*−Δr, *r*] as *N*_obs_(*i*, *j*, *r*), where *r* = 3, 4, 5, 6, 7, 8, 9, and 10 (Angstrom), and Δr is set as 3 for *r* = 3 and 1 for the rest of the values of *r*. In this study, the number of pairs of atom types *i* and *j* with the distance falling within a specified range, *N*_obs_(*i*, *j*, *r*), are calculated based on the protein-DNA complex structures collected from PDB. A complex is selected if a) it is an X-ray structure with resolution better than 3.0 Å, b) it contains exactly one double-strand DNA (dsDNA), c) the DNA molecule has ≥6 paired bases and has less than 30% non-paired bases, d) one of the protein chains has ≥5 contact residues (residues within 4.5 Å to the DNA molecule), and e) at least one of the protein chains has a length ≥40. Based on the PDB release of 25 October 2009, 549 protein-DNA complexes, containing 791 protein chains, satisfy all the criteria listed above. With *N*_obs_(*i*, *j*, *r*) of all the combinations, the potential between atom types *i* and *j* is represented as follows:
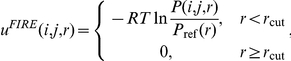
where *P*(*i*, *j*, *r*) = *N*_obs_(*i*, *j*, *r*)/Σ*_r_N*_obs_(*i*, *j*, *r*), *P*_ref_(*r*) = *r^α^*Δ*r*/Σ*_r_r^α^*Δ*r*, *r*_cut_ = 10 Å, and *α* is set as 1.61 as suggested in [12]. In the proposed method, we further improve the performance of the FIRE function by employing a distance-dependent weighting scheme to emphasize the influence from long-distance contacts. That is, 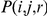 = *w*(*r*)×*N*_obs_(*i*, *j*, *r*)/Σ*_r_N*_obs_(*i*, *j*, *r*). For a given complex, the binding free energy, Δ*G*, is defined as the sum of all the potentials of the observed atom pairs [10]:
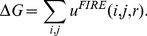
(1)


Assume that influences on binding free energy from different positions are independent, and thus Δ*G* can be represented as follows:
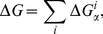
(2)where 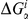 is the binding free energy of a base *α* (A, T, C, or G) at position *i*. By combining Eq. (1) and (2), we can estimate the probabilities in each column of PWMs as follows:
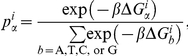
where *β* is a free parameter. The value of *β* was set as 15 in this study. It was chosen according to the performance of the proposed method on the 13 cases in Table 1 that were not included in the validation set.
